# Bioactive Polyurethane Shape Memory Polymer Foam Dressings with Enhanced Blood and Cell Interactions for Improved Wound Healing

**DOI:** 10.1021/acsami.5c02532

**Published:** 2025-04-22

**Authors:** Natalie Marie Petryk, Nghia Le Ba Thai, Leo Vikram Saldanha, Shawn Tyrin Sutherland, Mary Beth B. Monroe

**Affiliations:** Biomedical and Chemical Engineering and BioInspired Syracuse: Institute for Material and Living Systems, Syracuse University, Syracuse, New York 13244, United States

**Keywords:** collagen, gelatin, hemostatic dressing, smart biomaterials, traumatic wound healing

## Abstract

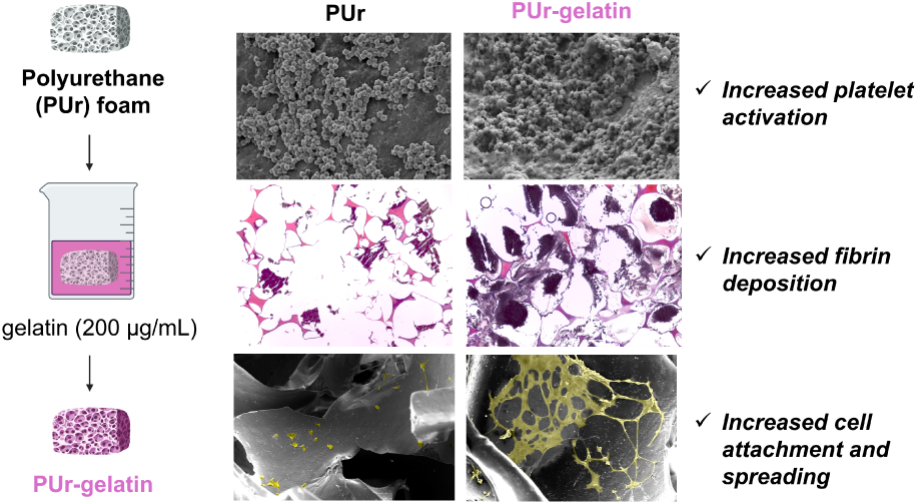

Polyurethane (PUr) shape memory polymer (SMP) foams have demonstrated excellent bleeding control in traumatic wounds. Unlike the current clinically available treatment options, PUr SMP foams can address noncompressible bleeds, are safe for prolonged use, and are highly tunable, offering broad functionalities like biodegradation and antimicrobial properties. Despite their hemostatic efficacy, PUrs are entirely synthetic, which limits their long-term healing capacity if left in a wound to degrade. This work employed methods for facile incorporation of bioactive collagen and gelatin into PUr foams postfabrication to enhance their clotting efficacy and drive cell interactions. The procoagulant nature of collagen and gelatin increased the clotting accomplished by the PUr SMP foams. Additionally, the bioactive PUr SMP foams promoted cell attachment, spreading, and proliferation on foam pores, which could facilitate tissue migration into the scaffold and promote wound repair. Overall, a bioactive PUr SMP foam dressing could significantly improve traumatic wound healing outcomes.

## 1 Introduction

Polyurethanes (PUrs) are versatile polymers with broad biomedical applications due to their biocompatibility, durability, flexibility, resistance to tearing and abrasion, and elastomeric properties.^1,2^ PUrs have been used across many areas of medicine, including cardiovascular grafts, catheters, balloons, and stent coatings; orthopedic cancellous bone substitutes, ligament reconstruction, and meniscus replacements; and reconstructive breast implants, wound dressings, and tissue adhesives.^3,4^ PUr shape memory polymer (SMP) foams are a special class of PUrs that offer unique biomedical applications. They are “smart” materials that can undergo a volumetric expansion in the presence of an external stimulus, such as temperature, pH, enzymes, light, electrical impulses, or magnetic induction.^5−8^ SMPs are synthesized in a primary shape and can be programmed into a temporary, secondary shape upon applying an external stimulus. A second stimulus can trigger recovery back to its primary shape, which may or may not be the same as the stimulus used for programming.^8,9^

**Figure 1:**
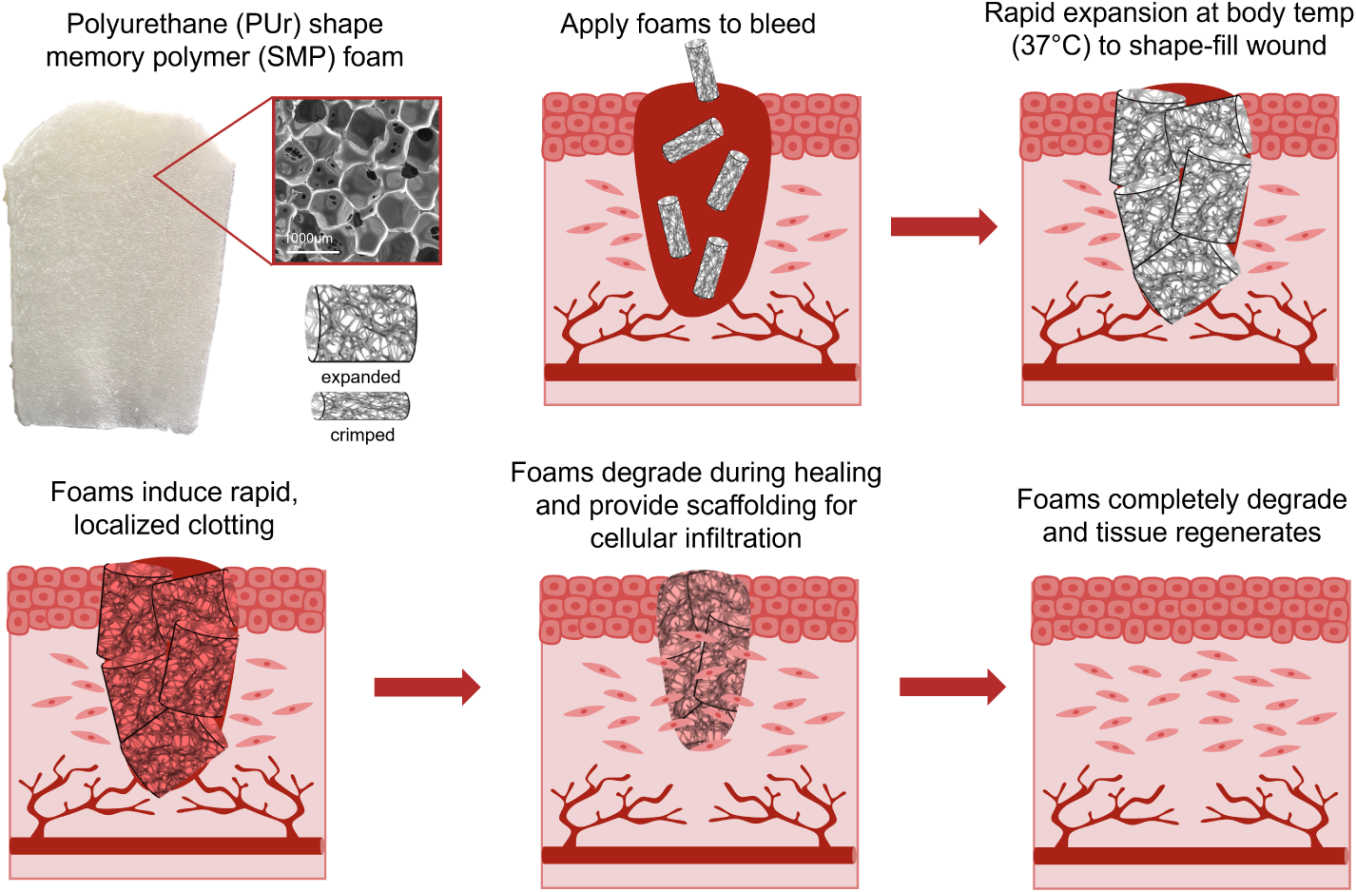
Intended application of PUr SMP foams to achieve hemostasis in a traumatic wound and facilitate tissue remodeling.

Thermally activated PUr SMP foams can be synthesized in their open-porous, primary shape. They can be programmed into a secondary shape by heating above their glass transition temperature (Tg) and deforming them into a low-profile, crimped shape. Once cooled back to below their Tg, they will remain in this secondary shape until exposed to a second stimulus.^10,11^ This unique capability makes PUr SMP foams advantageous in embolic applications and bleeding control. For example, PUr SMP foams can be used to treat aneurysms.^12^ In their low-profile, secondary shape, they can be delivered through a catheter to the aneurysm. The recovery rate is controlled so that only upon reaching the injury site will the foam recover to its primary, expanded shape after exposure to body temperature blood (thermal activation above its water-plasticized (wet) Tg). Then, they can shape-fill the ballooning vessel to occlude blood flow and redirect flow around the injury site.^13^ Similarly, PUr SMP foams can be used as a hemostatic dressing to control bleeding in traumatic wounds.^14−17^ They can be stored in their dry, low-profile secondary shape. Upon injury, they can be delivered to a bleed, where they rapidly expand to shape-fill an irregularly shaped wound when exposed to body temperature blood and induce localized clotting (Figure 1).^14^

PUr SMP foam hemostatic dressings have many advantages over commercially available treatment options: they are biocompatible and hemocompatible; their high porosity and low density allow for high blood absorption and minimal pressure applied to the surrounding tissue; their shape-filling property allows for their application into deep, irregularly shaped wounds; and they can address noncompressible wounds, initiating rapid, localized clotting.^11,14,15^ PUr SMP foams have demonstrated increased clotting efficacy compared to QuikClot Combat Gauze (QCCG) and XStat clinical treatment options, resulting in higher survival rates in a lethal porcine liver injury.^14^

Furthermore, PUrs are highly tunable, offering broad functionalities. They can be tailored to degrade to eliminate rebleeding upon dressing removal, a major limitation of currently available dressings.^16,18−21^ If left in a wound to degrade after stopping bleeding, these foams can serve as a scaffold for cell infiltration to promote tissue regeneration (Figure 1). Although PUr SMP foams are highly biocompatible, they are entirely synthetic, which inherently limits their ability to promote healing. In previous work, PUr scaffolds have been modified with bioactive components, such as chitosan, collagen, gelatin, hyaluronic acid, and RGD peptides, to drive desired biological responses.^22−26^ Incorporation of a bioactive component can facilitate all stages of traumatic wound healing, from hemostasis to tissue remodeling, to improve overall healing outcomes.

Here, we incorporated bioactive gelatin or collagen into our PUr SMP foams. Both gelatin and collagen are widely used in the design of hemostatic dressings and tissue engineering scaffolds because of their procoagulant properties and ability to promote cell attachment and proliferation.^27−29^ A hemostatic dressing should first and foremost control bleeding to stabilize the patient; procoagulant gelatin and collagen can enhance the bleeding control initiated by PUr SMP foams. Additionally, long-term use of a biodegradable dressing should offer scaffolding to promote new, healthy tissue. A bioactive PUr SMP foam could support cell attachment for tissue remodeling, which an entirely synthetic polymer scaffold could hinder. This work explores the physical and chemical incorporation of gelatin and collagen into PUr SMP foams to enhance healing processes without significantly altering the shape memory, mechanical, thermal, and structural properties that already make these hemostatic dressings effective at controlling bleeding in traumatic wounds. In the long term, this approach could be used to improve overall traumatic wound healing outcomes.

## 2 Methods

### 2.1 Materials

*N*,*N*,*N*′,*N*′-tetrakis(2-hydroxy-propyl)-ethylenediamine (HPED), triethanolamine (TEA), hexamethylene diisocyanate (HDI), and dibutyltin dilaurate (DBTDL) were purchased from Fisher Scientific (Waltham, MA) and used as received. 1,4-diazabicyclo[2.2.2]octane (DABCO 33 LV) was purchased from Sigma-Aldrich (St. Louis, MO) and used as received. Vorasurf DC 198 surfactant was provided by DOW (Midland, MI). Phosphate-buffered saline (PBS), Dulbecco’s phosphate-buffered saline (DPBS), gelatin (type A, 175 bloom), methacrylic anhydride, Dulbecco’s modified Eagle’s medium (DMEM), penicillin–streptomycin (P/S), and fetal bovine serum (FBS) were purchased from Thermo Fisher Scientific (Waltham, MA) and used as received. TeloCol-3 Type I Collagen Solution, 3 mg/mL (bovine) was purchased from Advanced Biomatrix. Na-citrated whole porcine blood was purchased from Lampire Biological Laboratories (Pipersville, PA). Lithium phenyl-2,4,6-trimethylbenzoylphosphinate (LAP) photoinitiator, glutaraldehyde (25%), and formaldehyde (≥36%) were purchased from Sigma-Aldrich (St. Louis, MO) and used as received.

### 2.2 Foam Synthesis

**Figure 2:**
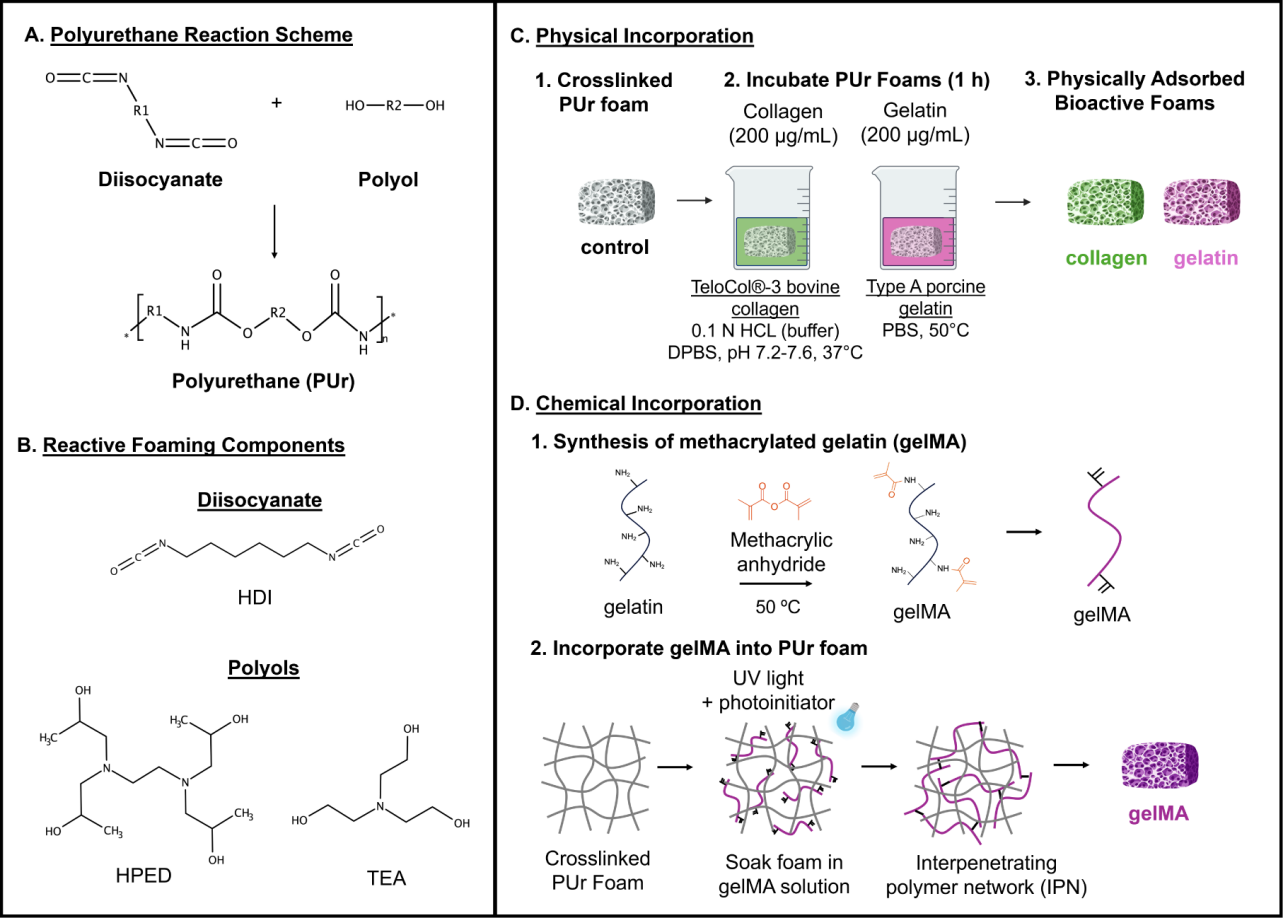
Overall reaction scheme for (A) the PUr foam synthesis and (B) its specific reactive foam components. Incorporation of the bioactive components through (C) physical incorporation and (D) chemical incorporation.

PUr foams were made as previously described (Figure 2a,b).^16,17^ A 16 g isocyanate (NCO) premix (1 NCO mol equivalent [HDI] and 0.35 hydroxyl (OH) mol equivalents [HPED and TEA]) was combined inside a controlled atmosphere glovebox, mixed at 3500 rpm for 30 s in a high-speed mixer (FlackTek, Landrum, SC), and then placed in a 50 °C oven to react for 48 h. After 48 h, the premix was cooled to room temperature, Vorasurf DC 198 surfactant was added, and it was mixed at 3500 rpm for 30 s. An 8 g OH mix was prepared with the remaining 0.65 OH mol equivalents (HPED and TEA), deionized (DI) water, and catalysts (DBTDL and DABCO 33 LV) by mixing at 3500 rpm for 30 s. The OH mix was combined with the NCO mix, speed-mixed at 1800 rpm for 5 s, quickly poured into a 400 mL cylindrical mold, and transferred to a 50 °C oven to foam for 5 min. The wt % of each reactive foaming component was 54.01, 27.61, 8.05, and 2.37 for HDI, HPED, TEA, and DI water, respectively. Catalysts and surfactant were added at 0.23, 0.37, and 7.36 wt % for DBTDL, DABCO, and Vorasurf, respectively. After polymerization, foams were washed with DI water and 70% ethanol to remove catalysts and surfactant and then dried under vacuum before characterization and analysis.

### 2.3 Physical Incorporation of Gelatin and Collagen

PUr foams (∼20 mg) were sterilized in 70% ethanol for 1 h and then incubated in DI water overnight to fully remove the ethanol. The samples were then placed in a sterile biosafety cabinet and coated in collagen following the TeloCol-3 collagen recommended coating procedure. Briefly, a 200 μg/mL solution was prepared by gently mixing sterile Type 1 collagen, DPBS, and sterile 0.01 N HCl buffer solution to achieve a final pH of 7.2–7.6. Foam samples were incubated in the collagen solution for 1 h at 37 °C (Figure 2c). PUr foams were similarly coated in a 200 μg/mL gelatin solution. Gelatin was dissolved in DPBS by heating the solution to 50 °C under constant stirring at 300 rpm. After dissolution, foam samples were submerged in the solution and incubated for 1 h at 37 °C (Figure 2c). After 1 h, the collagen- and gelatin-coated samples were removed from their solutions and rinsed with sterile PBS. Samples were further sterilized before cell studies using a UV box (254 nm) for 30 min.

### 2.4 Chemical Incorporation of Methacrylated Gelatin

Methacrylated gelatin (gelMA) was synthesized to chemically incorporate gelatin into PUr foams (Figure 2d).^30^ Type A porcine gelatin was added at 10% w/v to DPBS and mixed in an oil bath at 60 °C under constant stirring (500 rpm). After complete dissolution, the temperature was reduced to 50 °C, and 1.25% v/v methacrylic anhydride was added at 0.5 mL/min and allowed to react for 1 h. DPBS warmed to 40 °C was added at 2× volume to stop the reaction. Then, the solution was dialyzed for 3 days using 3.5 kDa molecular weight cutoff dialysis tubing to remove salts and unreacted methacrylic acid. The solution was frozen at −80 °C for 24 h before lyophilizing for 3 days. The resulting gelMA was stored in an −80 °C freezer for later use.

GelMA was dissolved in DPBS at 0.08 mg/mL at 60 °C, and then 1 mg of LAP photoinitiator was added and quickly combined using a vortexer. Sterile 20 mg foam samples were placed in a 24-well plate and incubated in 1 mL of the gelMA solution for 5 min. After 5 min, the foams were removed from the solution and gently pressed to remove excess gelMA. The samples were returned to a dry 24-well plate and placed in a UV light box (365 nm) for 3 min to cure the gelMA (Figure 2d).

### 2.5 Spectroscopic Analysis

The surface chemistry of each foam was characterized using a Nicolet i70 attenuated total reflectance Fourier transform infrared (ATR-FTIR) spectrometer (Fischer Scientific, Waltham, MA) to confirm the physical and chemical incorporation of the bioactive components. Small foam pieces (∼2 mg) were scanned at a resolution of 4 cm^–1^. Then, OMNIC software was used to generate spectra of each sample presented as absorbance vs wavelength over an average of 16 scans.

### 2.6 Pore Structure Analysis

Dry foam samples (∼1 cm^3^, *n* = 3) were coated in gold for 45 s using a high vacuum sputter coater (Denton Vacuum, Moorestown, NJ). A JEOL JSM-IT100 scanning electron microscope (SEM, JEOL USA, Peabody, MA) was used to image pore morphology at 10 kV and 25× magnification.

### 2.7 Density

Foam densities were measured in their primary (expanded) and secondary (crimped) shapes. Cylindrical foam samples (*n* = 3, ∼2.5 cm length, 8 mm diameter) were weighed. The volume in their expanded, open-porous shape was calculated from each length and average diameter. Density was calculated from the mass and volume. To determine density in their low-profile, crimped shape, the same foams were heated to 70 °C for 10 min, manually crimped radially along their length under constant load (Blockwise Engineering, Tempe, AZ), and cooled to room temperature to program into their secondary shape. Then, volume was measured, and density was calculated.

### 2.8 Thermal Analysis

The glass transition temperature (Tg) of dry foam samples (*n* = 3, 3–5 mg) was measured using a DSC 250 differential scanning calorimeter (DSC, TA Instruments, New Castle, DE). Samples were placed in T-zero aluminum pans, equilibrated at −40 °C, heated to 120 °C at 10 °C/min, held isothermally for 2 min, cooled to −40 °C at 10 °C/min, and then heated to 120 °C at 10 °C/min. The Tg was determined from the half-height transition of the second heating cycle. The Tg of wet, plasticized foams (*n* = 3, 3–5 mg) was measured by preparing samples in DI water at 50 °C for 10 min. After plasticizing, the samples were pressed dry, fitted into T-zero aluminum pans with hermetic lids, equilibrated at −60 °C, and then heated to 80 °C at 10 °C/min. The Tg was determined from the half-height transition of the single heating cycle.

### 2.9 Shape Memory Properties

#### 2.9.1 Shape Fixity

After measuring the volume of the crimped samples described above (0 h postcrimping), the samples were stored in 20 mL scintillation vials to keep them dry overnight. After 24 h postcrimping, each foam’s dimensions were measured again. The 24-h shape fixity of each foam was calculated as
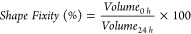


#### 2.9.2 Shape Recovery

After measuring shape fixity, the same foam samples were heated to 70 °C for 5 min to re-expand to their primary shape. Then, a 300 μm nitinol wire was threaded axially through each sample. The foams were reheated for 10 min, radially crimped as described above, and length and average diameter were measured for the wire-threaded foams. The wire was used to fix each sample to a metal plate to keep the foams submerged under water. The foams were placed in a 37 °C water bath for 5 min, and a camera was used to record the radial volume expansion (images taken every 5 s). The length and diameter of the final expanded volume were measured. ImageJ was used to measure dynamic changes in length and diameter from the recorded images. The shape recovery profile was determined as percent volume recovery over time, compared to the primary expanded volume.

### 2.10 Swelling Ratio

The dry mass (W_d_) of cylindrical foam samples (*n* = 3, ∼1 cm length, 8 mm diameter) was measured. Then, each sample was incubated in DI water for 24 h. After 24 h, the samples were gently patted to remove excess water, and the swollen mass (W_s_) was measured. Swelling ratio (SR) was calculated as
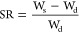


### 2.11 Mechanical Testing

The compressive modulus was measured for both dry and wet cylindrical samples (*n* = 3, 8 mm diameter, 4 mm thickness). For each test, the strain rate was controlled at −1 mm/min and the end limit was set to 24 N. Dry samples were placed on the lower platen, the upper platen was lowered until just touching the sample, position and force were zeroed, and the test began. Runs were manually stopped before the upper platen contacted the bottom platen, or automatically when the end limit was reached. Stress (kPa) and strain (mm) were calculated from load (N), sample area (mm^2^), position (mm), and sample length (mm) measurements outputted from the test program. Compressive modulus was determined from the slope of the linear region of the stress vs strain curve between 0.02 and 0.1 strain. To measure compressive modulus of wet (plasticized) samples, samples were prepared by incubating in DI water at 50 °C for 20 min. Then, each sample was gently patted to remove excess water before testing as described above.

### 2.12 Blood–Material Interactions

#### 2.12.1 Prothrombin Generation

A Pig Prothrombin ELISA kit (Abcam, Cambridge, United Kingdom) was used to test prothrombin generation induced by foam samples (*n* = 3, 5 mg) and QuikClot Combat Gauze (QCCG, clinical control). Prothrombin standards were prepared according to the manufacturer’s guidelines. Foam samples were placed in a 96-well plate and incubated in Na-citrated platelet-poor plasma (PPP, 100× dilution) for 1 h at 37 °C. After 1 h, the samples were transferred to the ELISA microplate and incubated for 2 h at room temperature. The plate was washed with buffer, treated with 1× Biotinylated Prothrombin Antibody for 1 h, and then rewashed and treated with 1× Streptavidin-Peroxidase Conjugate for 30 min. The plate was washed again, treated with chromogen substrate for 10 min, and the stop solution was added. A microplate reader (BioTek Synergy 2, Agilent Technologies, Winooski, VT) was used to measure the absorbance of each sample at 450 nm. The prothrombin standards were used to plot a standard curve of optical density (450 nm) vs prothrombin concentration (0 to 400 ng/mL), then fit to a logarithmic curve. The prothrombin concentration of each sample was calculated from the curve.

#### 2.12.2 Platelet Attachment

A lactase dehydrogenase (LDH) cytotoxicity assay kit (Cayman Chemical, Ann Arbor, MI) was used to quantify platelet attachment to the foams and QCCG. Na-citrated whole porcine blood was centrifuged at 10,000 rpm for 7 min to isolate platelet-rich plasma (PRP). The PRP was diluted with PBS to obtain standards (100, 50, 25, 12.5, and 6.5% PRP), and a hemocytometer was used to count the platelet concentration of each standard (*n* = 3). PUr foams (*n* = 3, ∼20 mg) were added to a 24-well plate with 1 mL of whole blood. After incubating at 37 °C for 30 min, the samples were gently rinsed with PBS to remove nonattached platelets. Then, the samples were transferred to a new 24-well plate with 1 mL of fresh PBS and 100 μL of 10% Triton X-100 to lyse the attached platelets at 37 °C for 1 h. Then, 100 μL of sample supernatant or 100 μL of each standard was transferred to a 96-well plate, and 100 μL of the LDH reaction solution (prepared following the LDH assay protocol) was added to each of the wells. The plate was incubated for 30 min at 37 °C. Then, the absorbance values were read at 490 nm using a microplate reader (BioTek Synergy 2, Agilent Technologies, Winooski, VT). The average platelet count on each sample was determined from a standard curve based on the absorbance of each standard versus the hemocytometer platelet counts.

#### 2.12.3 Clotting Time

Foam samples and QCCG (*n* = 4, ∼5 mg) were placed in the tip of 1.5 mL centrifuge tubes. Calcium chloride was added to Na-citrated whole porcine blood to obtain a 1 mM calcium chloride concentration and reverse the anticoagulant. Then, 50 μL of the recalcified blood was added to the samples and blank tubes (negative control). The samples were incubated in the blood at room temperature for 0, 6, 12, and 18 min, after which 1 mL of DI water was added to each tube for 5 min to lyse any free (unclotted) red blood cells (RBCs). The tubes were centrifuged at 10,000 rpm for 5 min, and then 200 μL of lysate was collected from each tube and added to a 96-well plate. A microplate reader (BioTek Synergy 2, Agilent Technologies, Winooski, VT) was used to measure the amount of hemoglobin released from the lysed RBCs at an absorbance of 540 nm.

#### 2.12.4 Clotting under Dynamic Blood Flow

##### 2.12.4.1 Direct Perfusion *In Vitro* Flow Model

**Figure 3:**
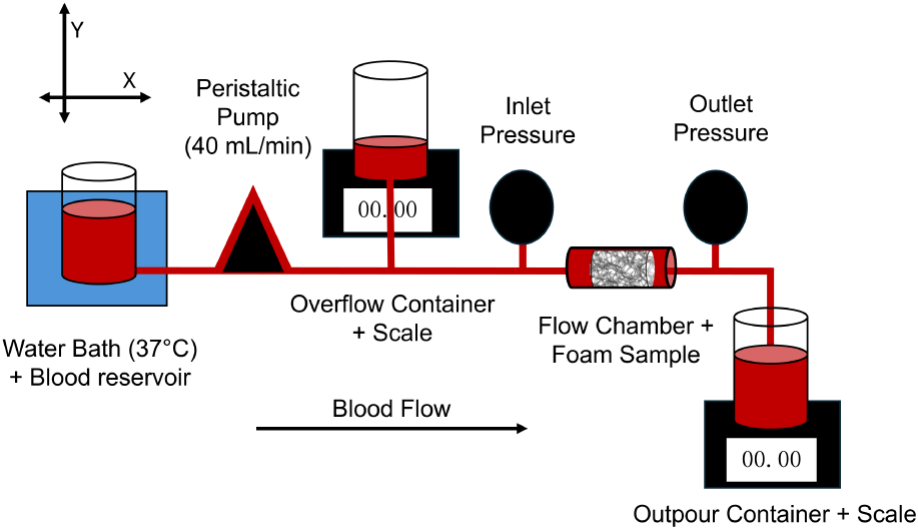
Schematic representation of the direct perfusion model built and used to test dynamic clotting behavior of the PUr foams.

A direct perfusion model shown in Figure 3 was built to study the effects of the bioactive components on foam-induced clotting under dynamic blood flow *in vitro*.^31^ A peristaltic pump (Thermo Scientific FH100 Peristaltic Pump, Fisher Scientific) was used to control blood flow at 40 mL/min through the flow system. Foam samples were loaded into a flow chamber, and digital high-accuracy pressure gauges (0–5 psi, McMaster-Carr, Elmhurst, IL) were fitted on either side. A 1 L “outpour” container (Pyrex borosilicate glass bottle, fitted with DWK Life Sciences DURAN Screw Cap 2-hose connector, Fisher Scientific) was connected at the end of the test section to collect blood that passed through the sample so that it would not recirculate through the system. A 1 L “overflow” container was connected before the first pressure gauge to collect blood that was rerouted due to a pressure increase and clotting. The outpour container was positioned below the flow system so that the tubing remained level on the benchtop, while the reroute tubing directed flow vertically into the overflow container (Figure 3); this setup helped to bias the flow forward through the test system and only reroute flow in the event of pressure buildup/clotting. Both the outpour and overflow containers were on electronic balances (Bonvoisin Lab Scale, 5000 g × 0.01 g), and cameras (AKASO V50X) were used to monitor dynamic changes in fluid mass collected in each container over time. Similarly, a camera was used to monitor changes in inlet and outlet pressures over time. All tubing used in the flow system was Tygon PVC plastic (ISO 10993 certified, nonhemolytic, nonpyrogenic, nontoxic tubing, used in biological applications.^32,33^ Tubing passing from the blood reservoir through the peristaltic pump was 1/8” ID, 1/4” OD, while all remaining tubing through the loop was 7 mm ID, 9 mm OD.

Before each run, PBS was perfused through the flow loop to prime the tubing and fittings. Dry foam samples (*n* = 3, 8 mm diameter, 2.5 cm length) were weighed and then preloaded into PBS-primed sacrificial flow chambers (7 mm diameter tubing, 20 cm length) by radially crimping the foam along its length, inserting it into the center of the tube, and placing the tube into an oven at 60 °C to allow the foams to re-expand to 110% fill. Before each run, the sample-loaded tube was connected to the flow loop via straight connectors. Then, 500 mL of Na-citrated whole porcine blood was warmed to 37 °C using a water bath. Foams were perfused with the warmed blood at 40 mL/min for 10 min. After 10 min, the fluid volume in each container was measured. The sacrificial flow chamber tubing was cut proximal and distal to the foam sample. The samples were carefully removed from the tubing and weighed to determine their swollen weight. Then, the samples were gently rinsed with PBS to remove nonadherent cells and cut into 6 equal cylindrical cross sections along the length of the foam: 3 cross sections—1 from the proximal, middle, and distal end of the foam relative to the direction of flow—were fixed in 4% formalin for histological analysis, and the other 3 were fixed in 2.5% glutaraldehyde for platelet imaging using SEM. Before subsequent test runs, bleach was perfused through the system to remove blood, followed by PBS for priming.

##### 2.12.4.2 Platelet Imaging

After 24 h in 2.5% glutaraldehyde, the foam cross sections were dehydrated in increasing concentrations of ethanol (50%, 70%, 95%, and 100%; 30 min each), and then dried in a vacuum oven overnight. The dried samples were loaded onto SEM stubs, sputter-coated with Au at 45 mA for 60 s, and imaged using SEM (1000× magnification, 10 kV).

##### 2.12.4.3 Histological Analysis

Foam sections cut from the proximal, middle, and distal end of the foams were removed from 4% formalin, dehydrated in increasing concentrations of ethanol (70% to 100%), and cleared with Epredia Signature Series Clear-Rite 3 (Fisher Scientific, Hampton, NH) using the Epredia STP 120 Spin Tissue Processor (Epredia, Kalamazoo, MI). Then, samples were infiltrated with paraffin wax using the Epredia STP 120 Spin Tissue Processor and embedded into paraffin blocks using the Epredia HistoStar Embedding Workstation. The paraffin blocks were sectioned (10 μm thicknesses) on an Epredia HM 355S Automatic Microtome, placed on an Epredia Flotation Bath set to 40 °C, floated onto glass slides, and dried in a Premiere Slide Warmer XH-2004 (Avantor, Radnor Township, PA) at 40 °C. Dried slides were deparaffinized with xylenes, rehydrated, and stained with hematoxylin and eosin (H&E) and phosphotungstic acid hemotoxylin (PTAH). Slides were imaged with a Leica DM300 microscope using a 4× and 40× objective. Fibrin percentage was quantified for each field of view at 4× from an average of *n* = 6 images per cross-section (proximal, middle, and distal) for *n* = 3 foams by isolating the PTAH-stained fibrin using the color-select tool in GIMP.

### 2.13 Cell Interactions

#### 2.13.1 Cytocompatibility

NIH/3T3 Swiss mouse fibroblasts (ATCC–CCL92) were cultured using DMEM (high glucose GlutaMAX) supplemented with 10% heat-inactivated FBS and 1% P/S (Gibco) at 37 °C/5% CO_2_. A Zeiss Axiovert inverted microscope was used to assess cell morphology and ensure even cell distribution (∼100% confluency) in a culture flask prior to all cell studies. After confirming morphology and confluency, the culture media was removed, and cells were rinsed with sterile PBS and detached from the culture flask with 1× trypsin. The trypsinized cells were added to a 15 mL centrifuge tube with 10 mL media and centrifuged at 1000 rpm for 5 min. The resulting cell pellet was resuspended in culture media. Then, 10 μL of the suspension was combined with 10 μL trypan blue and added to a hemocytometer to measure cell concentration. Cells were seeded in a 24-well plate at 10,000 cells/mL (600 μL/well) and incubated for 24 h. After 24 h, sterile foam samples (*n* = 3, 6 mm diameter, 2 mm thickness) were placed into Transwell inserts (6.5 mm, 0.4 μm pore polyester membrane, Corning) and added to the cell-seeded wells. Empty inserts were used as positive (cytocompatible) controls. After 24, 48, and 72 h, the insets and cell media were removed and 600 μL of 10% alamar blue (resazurin) was added to each sample-containing well and control wells (*n* = 3, positive: cells; negative: no cells) and incubated at 37 °C for 2 h. Then, 150 μL from each well was transferred to a 96-well solid black polystyrene plate. A microplate reader (BioTek Synergy 2, Agilent Technologies, Winooski, VT) was used to measure fluorescence (excitation: 530/25; emission: 590/35; position: top 50%). Cell viability was calculated as
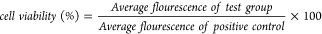


#### 2.13.2 Cell Attachment

To quantify cell attachment, 3T3 cells tagged with green fluorescent protein (GFP, Cell Biolabs Inc. AKR214) were cultured as described above, except the cell pellet after centrifugation was resuspended in reduced-serum media (DMEM, 2% FBS, 1% P/S) to control for cell attachment to foams due to serum protein adsorption. After counting cell concentration with the hemocytometer, the required volume (X) of cell suspension to achieve a final concentration of 50,000 cells/well was determined. This volume X was droplet seeded directly onto sterile foam samples (*n* = 3 for each time point, 6 mm diameter, 2 mm thickness) in a 96-well plate and set to incubate for 30 min at 37 °C. After 30 min, reduced-serum media was added at a volume of 200 - X μL to each well to reach a final concentration of 50,000 cells/well (200 μL/well) and returned to the incubator. Cell attachment was viewed on the samples using a Leica Thunder microscope after 24, 48, and 72 h. Z-stack images were acquired at 10× magnification, and ImageJ was utilized to determine the total cell count.

#### 2.13.3 Cell Spreading

Foam samples (*n* = 3 for each time point, 6 mm diameter, 2 mm thickness) were droplet-seeded as described above (Section 10.2) with standard 3T3 cells (no GFP). At each time point (24, 48, and 72 h), samples were removed from media and prepared for staining. The samples were rinsed 3 times with 1× PBS and fixed with 4% paraformaldehyde for 10 min. Samples were then washed with PBS 3 times for 5 min each before permeabilizing cells with 0.2% Triton X-100 for 20 min. Samples were again washed (PBS, 3 times, 5 min each) before blocking samples with blocking buffer (10% FBS, 1% BSA in 1× PBS) for 30 min. Phalloidin actin stain was added to blocking buffer (1:200), and samples were incubated with the stain at room temperature for 10 min. After 10 min, cells were washed with PBS (3 times, 5 min each). Then, DAPI in blocking buffer (1:2000) was added. After 5 min, the samples were rinsed with PBS. Finally, samples were imaged using an inverted microscope at 20× magnification.

The same samples were then prepared for SEM imaging by gently rinsing with PBS and fixing in 2.5% glutaraldehyde overnight at 4 °C. Then, the samples were dehydrated in ethanol (50%, 70%, 95%, and 100%; 30 min each) and dried in a vacuum oven overnight. SEM micrograph images were taken at 10 kV and 200× magnification to view cell morphology. The cells in each image were colorized using Adobe Photoshop. Then, GNU Image Manipulation Program (GIMP) was used to quantify cell spreading by using the color-select tool and the histogram analysis feature to determine the pixel area coverage of cells.

### 2.14 Statistical Analysis

Measurements are presented as mean ± standard deviations. A student’s *t* test was performed in Microsoft Excel with two-tailed distribution and two-sample unequal variance. If *p* < 0.05, differences were considered statistically significant.

## 3 Results

### 3.1 Incorporation of Bioactive Components

**Figure 4:**
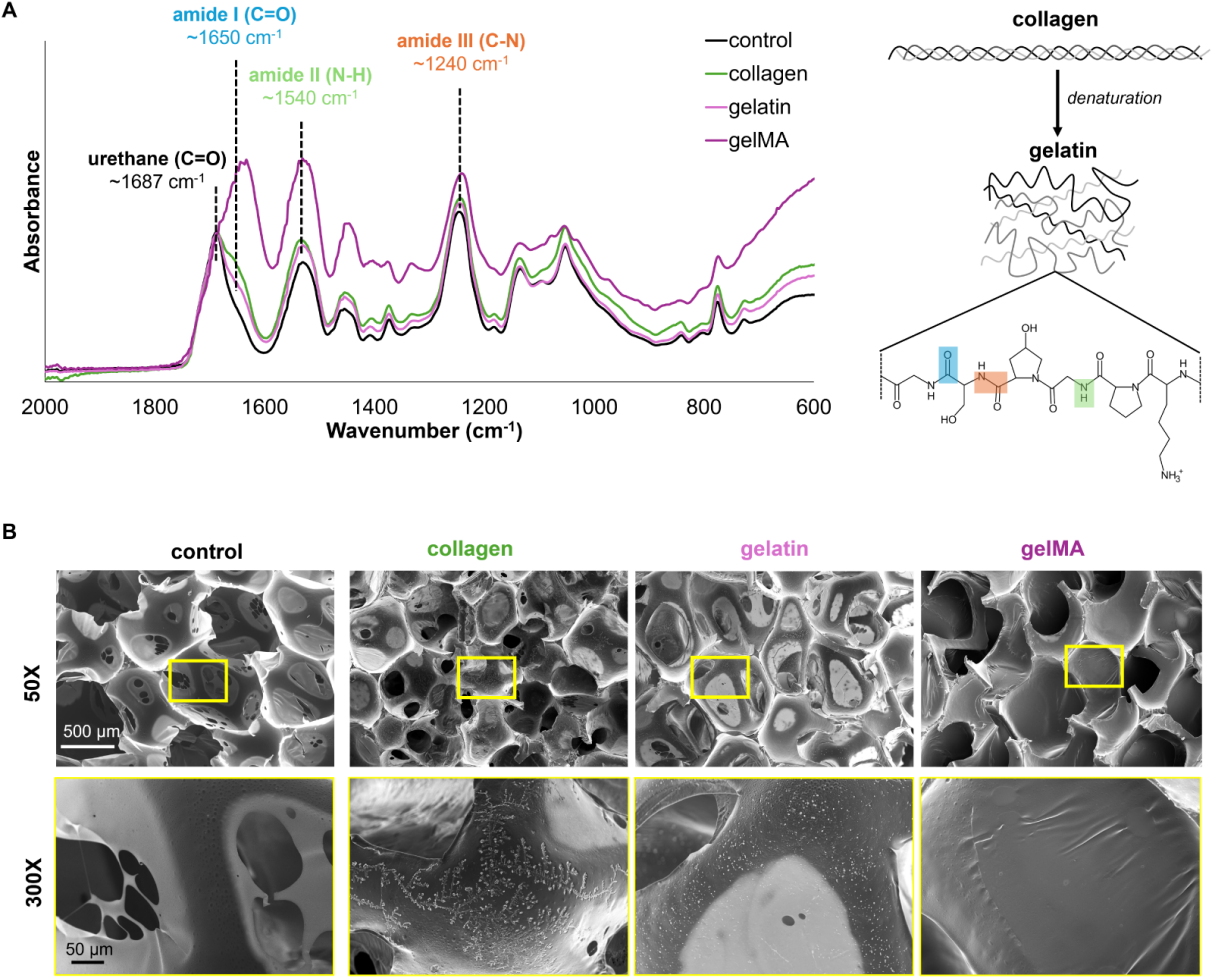
a) FTIR spectra confirming the incorporation of collagen, gelatin, and gelMA into the PUr foams based on amide peaks. b) SEM micrographs showing overall foam pore structure (50× magnification) and surface morphology (300X) of the bioactive foams compared to the control PUr.

Successful incorporation of collagen, gelatin, and gelMA is demonstrated by amide peaks in the FTIR spectra (Figure 4a). Each spectrum was normalized to have the same peak height of the C=O of urethanes (∼1687 cm^–1^), since the urethane quantity remains constant throughout each foam. The physically incorporated collagen and gelatin foam samples have a shoulder peak off of the urethane peak at ∼1650 cm^–1^ for amide I, while gelMA has a much stronger, fuller peak.^27,34^ The N–H and C–N of urethanes show strong peaks at ∼1540 cm^–1^ and ∼1240 cm^–1^.^35^ The increase in absorbance (peak height) among the bioactive foams at these peaks corresponds with the overlap of amide II and amide III, respectively.^27,34^

The control PUr SMP foam has relatively closed-cell pores (∼1000 μm diameter), with some pore interconnectivity due to the thinning of pore membranes during the gas-foaming fabrication process, seen by the lighter contrast areas and pinhole openings (Figure 4b). The physical incorporation of collagen and gelatin can be visualized in the SEM micrographs. Both collagen and gelatin are large proteins that are adsorbed to the surface of the pore membranes. The collagen appears as long, organized strands that branch off one another, while gelatin appears as less organized, bead-like structures on the pore surface (Figure 4b). In the gelMA foam composite, the pore surface appears smoother and the membranes more closed, suggesting that the cross-linked gelMA network is filling in the pores to a degree, resulting in less interconnected pores.

### 3.2 Foam Physical, Thermal, Shape Memory, and Mechanical Properties

**Figure 5:**
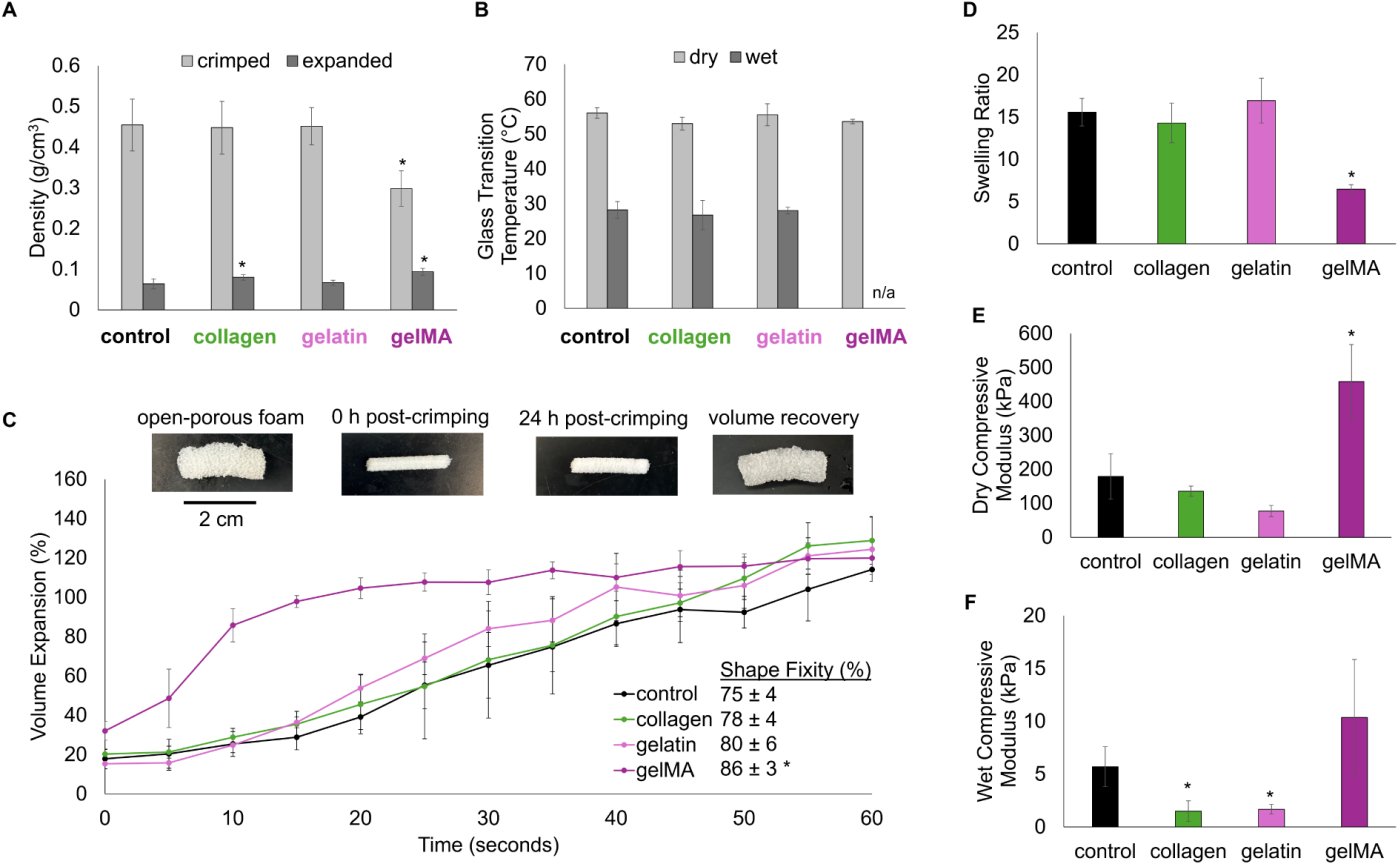
Foam characterization. A) Foam density in their expanded and crimped shapes. B) Glass transition temperatures of foams in their dry and wet (plasticized) state. C) PUr foam shape memory properties, including shape programming, fixing, and recovery (volume expansion in 37 °C water). D) Foam swelling ratios after 24 h in 37 °C water. E) Foam compressive modulus in their dry state. F) Foam compressive modulus in their wet (plasticized) state. *p < 0.05 relative to the control PUr foam.

The high porosity of these foams allows for low density in their open-porous (expanded) primary shape. This property provides a high surface area, resulting in high blood absorption and reduced pressure applied to surrounding tissue. The incorporation of collagen and gelMA into the foams significantly increases expanded foam density, an effect of the collagen fibers and gelMA network intertwined in the PUr foam pores (Figure 5a). Despite the increase in density, these foams are still considered low-density foams (<0.1 g/cm^3^). The ability to crimp these foams into a secondary, low-profile shape is important for storage and ease of delivery to a wound. The control, collagen, and gelatin foams all have comparable crimped densities (∼0.45 g/cm^3^), while the gelMA foam has a significantly lower crimped density ∼0.3 g/cm^3^. The gelMA network fills the otherwise open pores, making the foams denser and reducing the extent to which the foams can be compressed.

FTIR spectra were collected on the bioactive foam samples before and after 1-h incubation in DI water at 37 and 50 °C to ensure that bioactive components would not significantly dissolve during experiments where plasticized samples were tested (Supplemental Figure S1). All the foams had comparable glass transition temperatures (Tg) in their dry state, with Tg’s around 55 °C (Figure 5b). This property ensures good shape fixity of the foams in their low-profile shape in extreme conditions, like the high temperatures that may be reached in a battlefield setting. The control, collagen, and gelatin PUr foams all have wet Tg’s below 37 °C (Figure 5b). A wet Tg below body temperature is required to ensure plasticization and therefore rapid expansion when exposed to blood. The wet Tg of the gelMA sample could not be measured, because the gelMA network generated a large melting peak that consumed the Tg (Supplemental Figure S2).

Figure 5c shows an example of the control PUr foam in its primary synthesized shape, secondary crimped shape, fixed shape post 24 h, and recovered shape after expansion in 37 °C water. The incorporation of gelatin and collagen into the PUr foams increase their shape fixity, with the gelMA foam having a significant increase in shape fixity, improving foam maintenance of their crimped, low-profile shape (Figure 5c).

The control PUr is a thermally activated amorphous thermoset SMP foam that relies on hydrogen bonds to form and break during shape fixing and shape recovery. The amides found in gelatin and collagen introduce additional hydrogen bonding sites to the network, resulting in increased fixity.^36^ When the foams are exposed to body-temperature water, they are heated above their wet Tg and plasticize as hydrogen bonds within the network break and react with water. The gelatin- and collagen-coated samples have volume recovery profiles comparable to the control PUr (Figure 5c). The gelMA-foam composite has faster expansion, achieving 100% volume recovery in ∼20 s (Figure 5c). The gelMA network is most likely swelling, allowing the foam to recover its shape more rapidly.

The control, collagen, and gelatin foams all had comparable swelling ratios, having ∼150% increase in their absorbed weight (Figure 5d). The gelMA foam has significantly reduced swelling despite the swelling capacity of the gelMA hydrogel network. This result could be explained by the gelMA filling foam pores and creating a more closed-cell network as seen in Figure 4b, inhibiting water from accessing the bulk of the sample. Similarly, the control, collagen, and gelatin foams all have comparable compressive moduli in their dry state, whereas the gelMA foam has a significantly increased compressive modulus (Figure 5e). This increase in modulus is reflective of an increase in foam density. In its wet, plasticized state, the gelMA foam still has a higher average compressive modulus, but it is not significantly different than that of the control PUr. On the other hand, the collagen and gelatin foams had a statistically significant decrease in compressive moduli (Figure 5f).

### 3.3 Blood–Material Interactions

**Figure 6:**
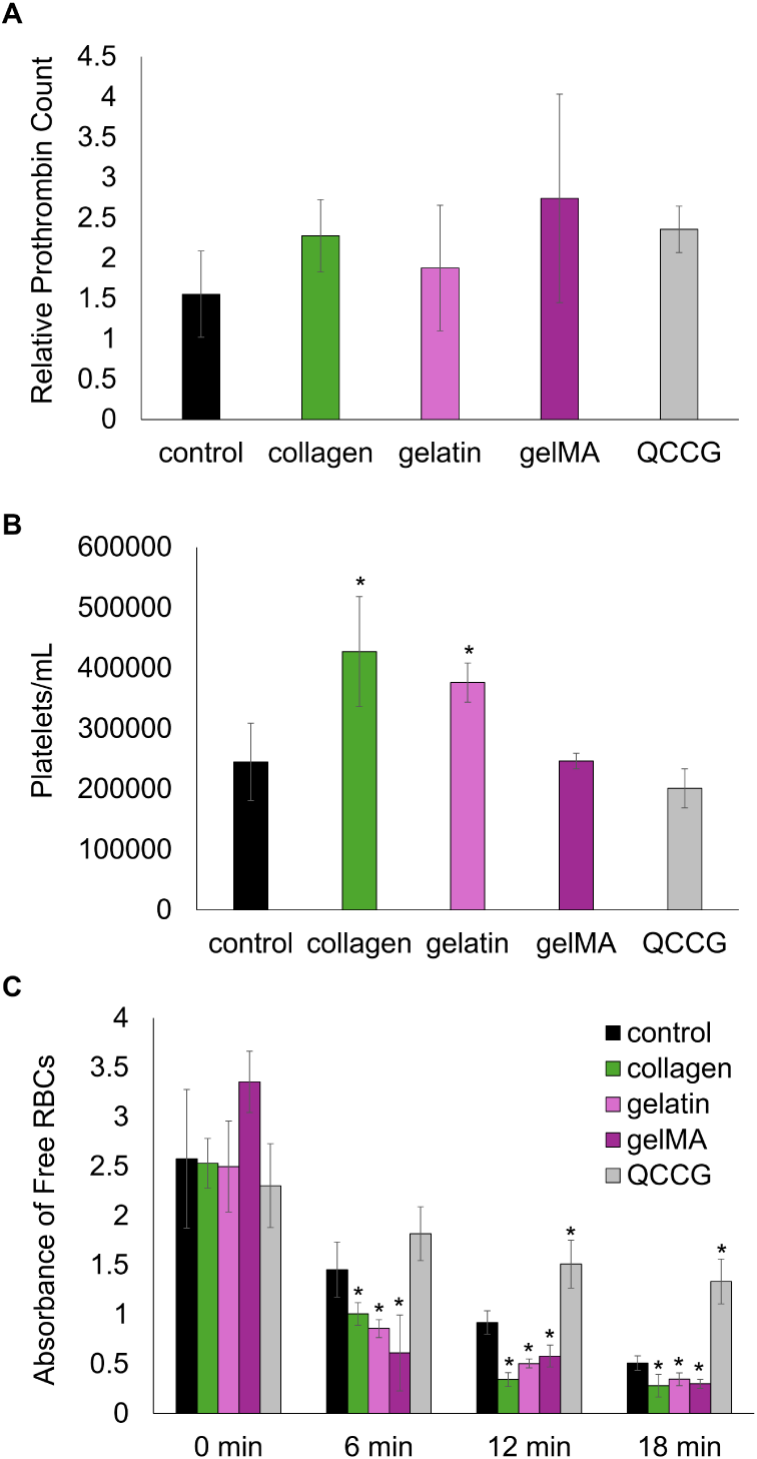
Foam clotting behavior under static conditions. A) Relative prothrombin count after 1 h incubation with Na-citrated whole porcine blood. B) Concentration of attached platelets to PUr foams and QCCG after 30 min incubation with Na-citrated whole porcine blood. C) Clotting time of PUr foams and QCCG based on the absorbance of free red blood cells (RBCs) collected in the lysate. **QCCG**: QuikClot Combat Gauze clinical control. *p < 0.05 relative to the control PUr foam.

The prothrombin generation of each foam was assessed under static conditions using ELISA. The control, collagen, gelatin, and gelMA foam samples all had comparable prothrombin generation with no statistically significant differences compared to the positive, clinical control, QCCG (Figure 6a). QCCG contains kaolin, a hemostatic agent that accelerates clotting. By generating prothrombin, the foams help initiate a series of chemical reactions that ultimately result in a fibrin mesh that helps to form a stable clot, providing a temporary seal to stop bleeding. The control PUr and gelMA foam had comparable platelet attachment to that of QCCG, while the collagen and gelatin foams had significantly higher platelet attachment (Figure 6b). Furthermore, the collagen, gelatin, and gelMA foams all induce clotting faster than the control PUr and QCCG. This result is demonstrated by the significant decrease in absorbance values of free RBCs in the lysate at 6, 12, and 18 min, compared to the control PUr and QCCG, indicative of more rapid clotting (Figure 6c).

**Figure 7:**
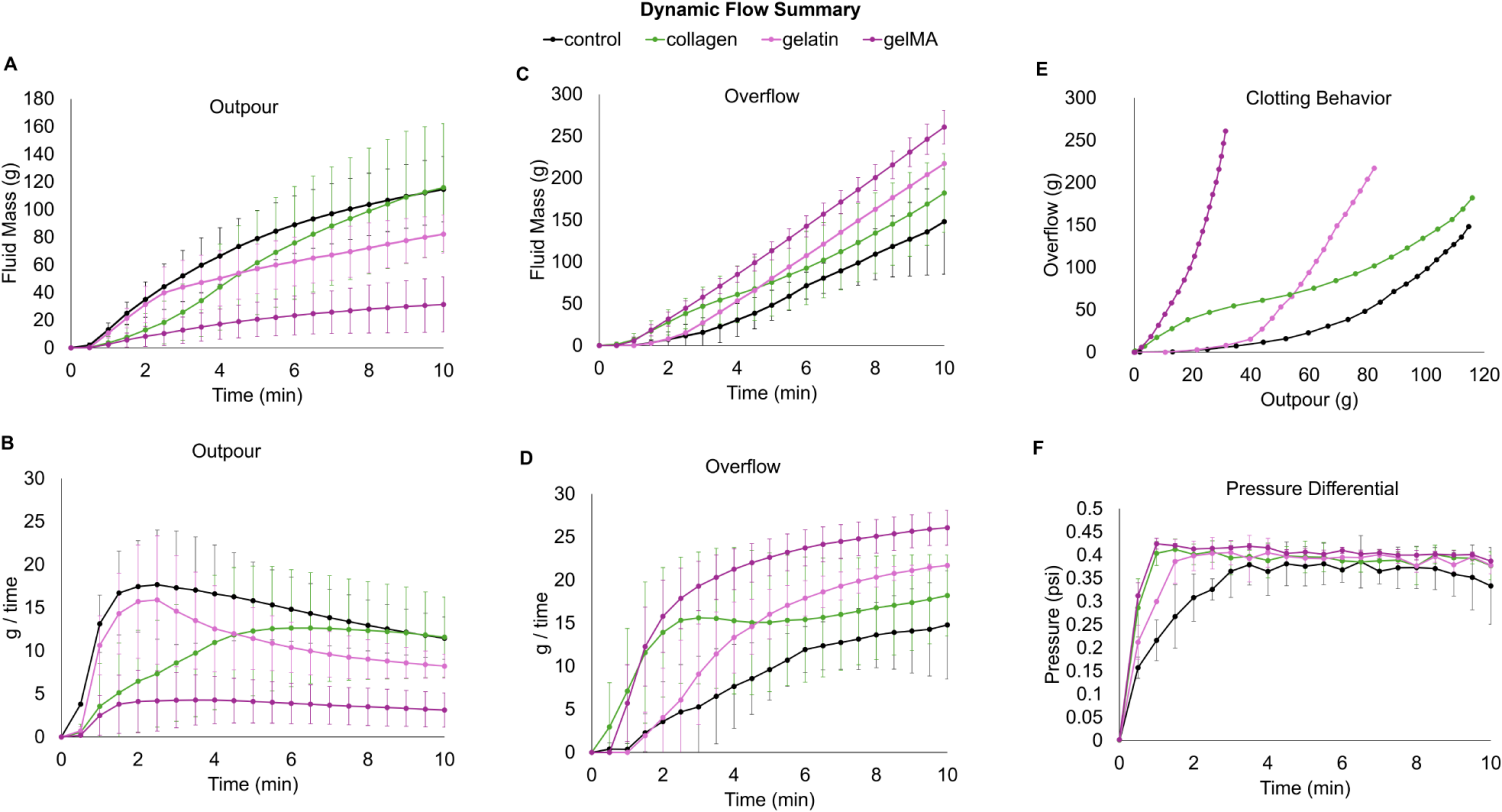
Dynamic clotting behavior of PUr foams. A) Fluid mass passed through each sample collected in the outpour container. B) Change in flow rates over time of fluid collected in the outpour container. C) Fluid mass rerouted into the overflow container. D) Change in flow rates over time of fluid collected in the outpour container. E) Overall clotting behavior of each foam based on rerouted blood versus blood through each sample. F) Pressure differential across each foam during each 10 min perfusion run.

Dynamic blood material interactions can help better predict how these foam-based hemostatic dressings may initiate clotting *in vivo*. The direct perfusion model designed for these studies offers an understanding of foam permeability and clotting based on dynamic measurements of the blood fluid mass that got rerouted or passed through the samples. Standard runs without any sample show that the blood is directed into the outpour container at a linear rate, with no rerouted flow (Supplemental Figure S3). For each sample, blood initially passes through the foams and is collected in the outpour container, with the highest flow rates within the first 2 min (Figure 7). Then, as clotting starts, the fluid mass in the outpour container starts to plateau (Figure 7a) and flow rates through the samples slow down (Figure 7b) as more blood starts to get rerouted. The fluid mass collected in the overflow container is relatively linear over time (Figure 7c). Figure 7e shows the overall clotting behavior of each foam, showing that all of the bioactive foams have increased clotting compared to the control, with more blood flow rerouted into the overflow container and less into the outpour. The gel foam had the greatest effect on redirecting flow. The bioactive foams reach a higher pressure differential across each foam faster, but by 4 min the pressure across each foam is comparable (Figure 7f).

**Figure 8:**
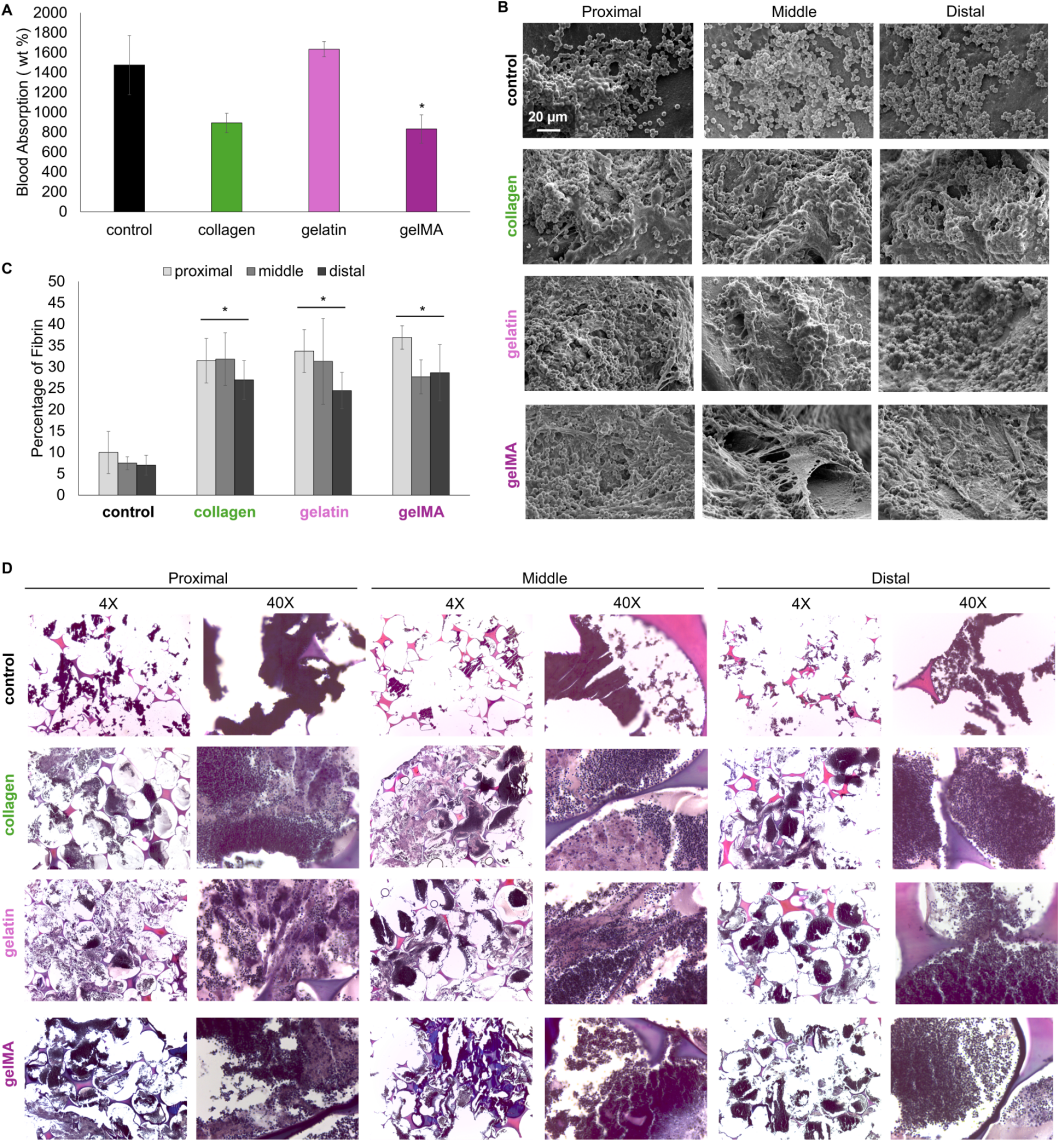
Blood-material interactions after 10 min of perfusion with Na-citrated whole porcine blood. A) Weight percent of blood absorbed during perfusion runs. B) Platelet interactions on foam pores. C) Fibrin quantification based on the PTAH-stained samples. D) Fibrin deposition on PUr foams at the proximal, middle, and distal end relative to the direction of blood flow. *p < 0.05 relative to the control PUr foam.

After perfusion with blood for 10 min, the swollen mass of the samples indicates that the control and gelatin foams adsorbed the most blood (Figure 8a), consistent with their swelling ratios in water in Figure 5d. An assessment of platelet interactions can further explain the flow behavior. Primary hemostasis is the body’s first response to injury, which starts with platelets adhering to damaged tissue.^37^ When vascular injury occurs, collagen is exposed and acts as a primary trigger for platelet adhesion and activation.^38^ Nonactivated platelets have a smooth, round morphology. Then, the platelets start to activate, changing morphology and exhibiting star-like protrusions; these activated platelets recruit more platelets to the injury site and form aggregates, creating a “platelet plug” to stop blood flow.^37^ Secondary hemostasis stabilizes the plug by introducing a series of clotting factors that lead to the formation of a fibrin mesh, creating a temporary seal.

The slightly hydrophobic surface of the PUr hemostatic foam dressings promotes platelet attachment, activation, and aggregation to facilitate primary hemostasis.^16,39^ The procoagulant nature of collagen and gelatin can enhance foam-induced clotting. After 10 min of perfusion with the anticoagulated blood, the control foam has platelets adhering throughout the bulk sample, as demonstrated through SEM micrographs taken at proximal, middle, and distal ends of the foam (Figure 8b). The platelets attached with some platelet activation. The aggregates formed are smaller, as the pore surface is still visible. The collagen, gelatin, and gelMA samples have increased platelet interactions throughout the length of each sample. There are a greater number of platelets attached, and the aggregates formed are larger, as demonstrated by little to no pore surface visible in each image (Figure 8b). There are more platelets activated on these foams, seen especially on the collagen sample, and at the distal end of the gelatin sample. Furthermore, there is fibrin deposition, as demonstrated by the strand and mesh-like structures, particularly in the gelMA samples (Figure 8b). In the gelatin and gelMA foam samples, the platelets appear nonactivated, but these platelets are trapped in a fibrin mesh, showing the efficacy of these foams in creating stable “plugs” capable of recruiting more platelets.

Histological staining of fibrin, a protein that stabilizes blood clots, provides further insight into the clotting behavior of each foam. Foam pore struts are stained pink by the eosin, while attached cells (red and white blood cells, along with other circulating cells from whole blood, such as endothelial cells, are stained a lighter purple by the hematoxylin. The fibrin is stained a dark purple by the PTAH, and its morphology can be seen in the higher magnification images where the fibrin appears as bead-like structures (Figure 8d). The control foam has significantly lower fibrin deposition than the collagen, gelatin, and gelMA samples throughout their bulk (Figure 8c). In all of the bioactive foams, fibrin can be seen throughout the sample volume, with the most fibrin present at the proximal end of each foam. SEM and histological images at the proximal, middle, and distal end of the foams show that blood is penetrating throughout the entire foam volume, maximizing the contact area for inducing clotting.

### 3.4 Cell Interactions

**Figure 9:**
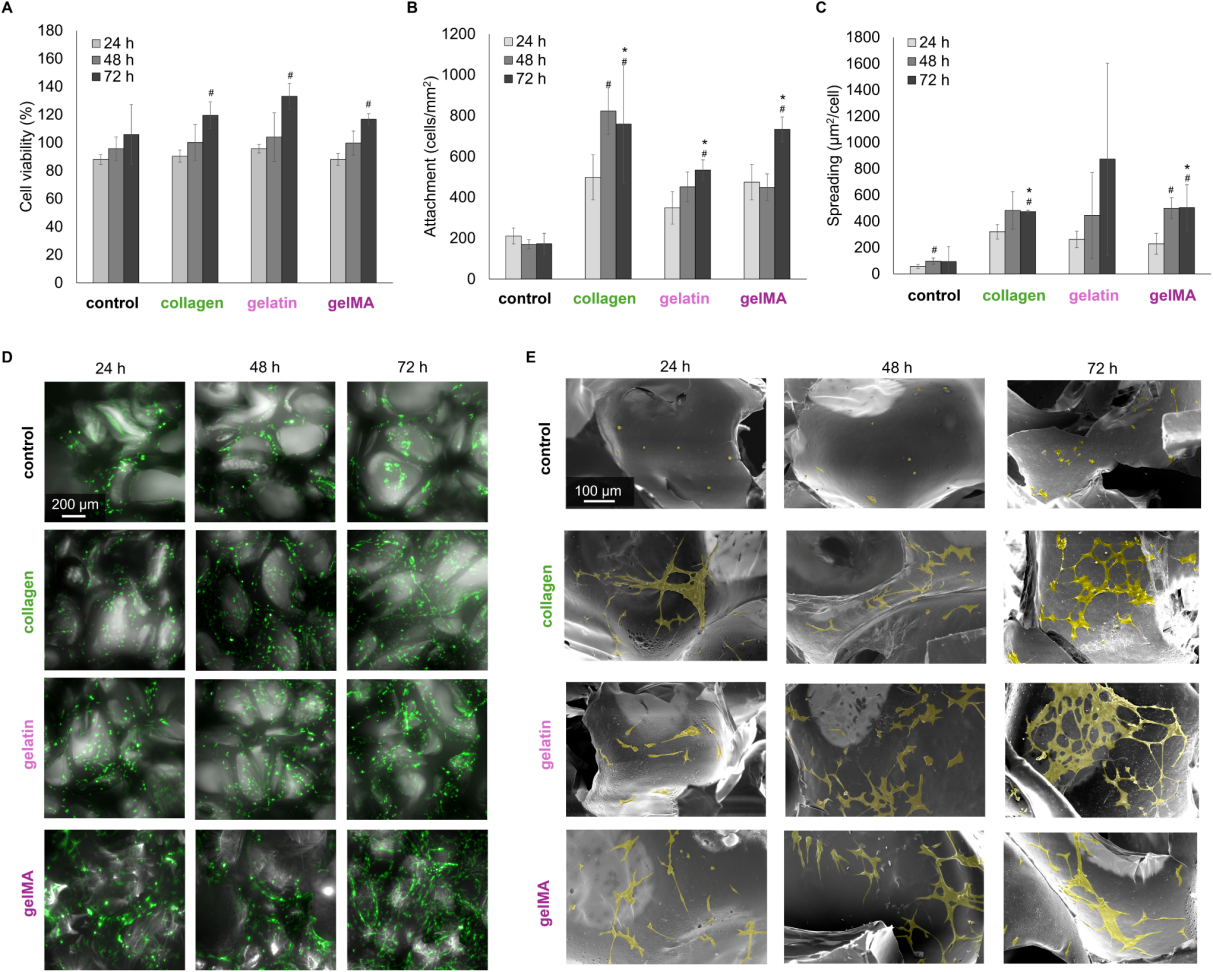
NIH/3T3 cell interactions on PUr foams. Changes in cell (A) viability, (B) attachment, and (C) spreading over 24, 48, and 72 h on PUr foams. D) Merged z-stack brightfield and GFP images of cells attached to PUr foams. E) SEM micrographs of cell spreading (colored yellow) on PUr foam pores. *p < 0.05 comparing 72 h time point of bioactive foams to that of the control; #*p* < 0.05 comparing 48 and 72 h time points to the 24 h time point within a sample.

All the foams are highly cytocompatible with cell viability >95% at 24 h, meeting the ISO 10993 biocompatibility standard of >75% viability for biomedical devices.^40^ Over 72 h, the bioactive foams have a statistically significant increase in cell viability compared to the control PUr (Figure 9a). Cells initially attach to the control PUr at 24 h, but the decrease in cell attachment over 48 and 72 h indicates that these cells are not proliferating and possibly dying (Figure 9b). The gelatin, collagen, and gelMA foam samples had a higher initial number of cells attached after 24 h, which continued to proliferate and spread through the 72 h time point (Figure 9b,c). The z-stack images of foam pores (brightfield) and cells (GFP) demonstrate that cells are attaching and proliferating throughout the sample (Figure 9d).

**Figure 10:**
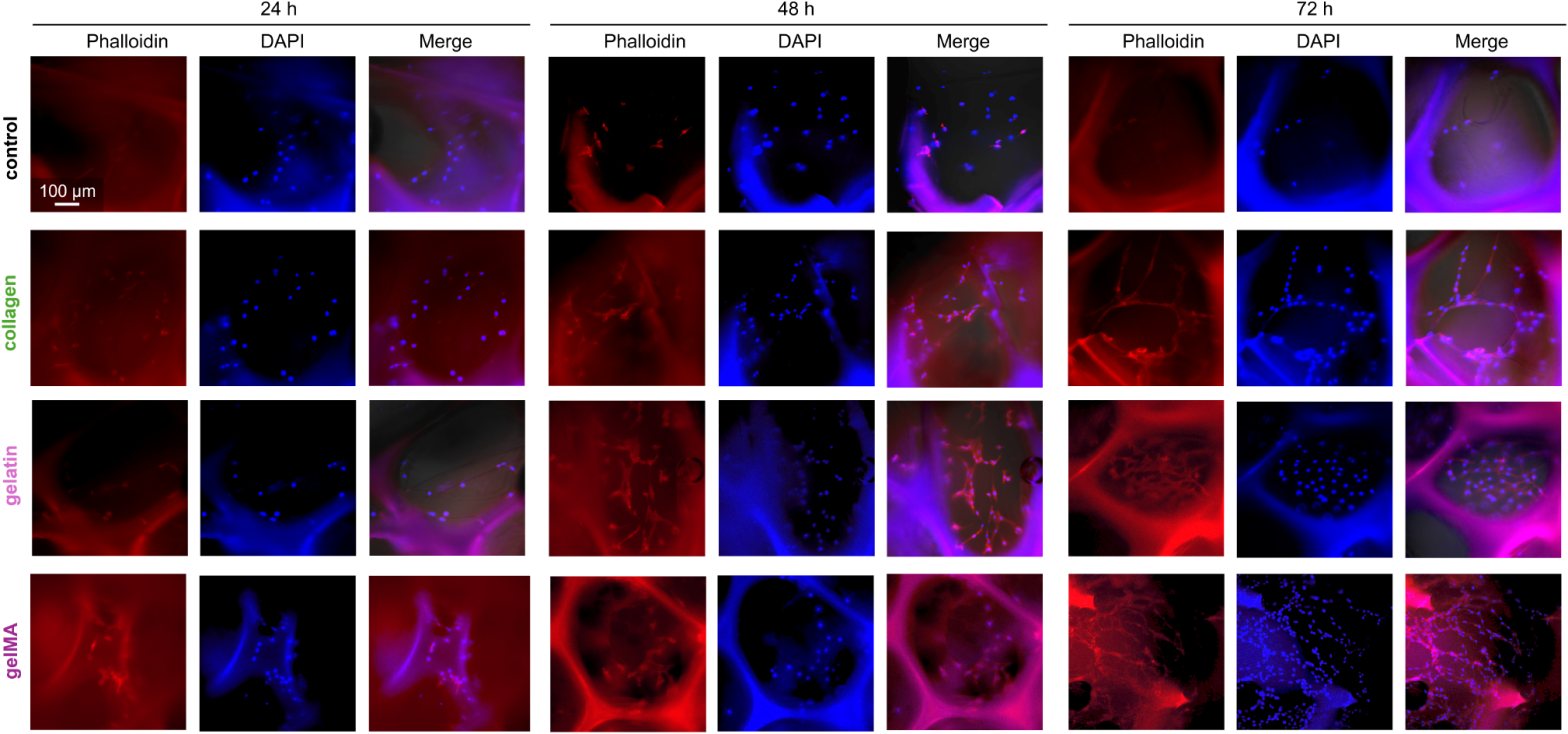
DAPI (nuclei, blue) and phalloidin (actin, red) staining of NIH/3T3 cells on foam pores after 24, 48, and 72 h.

SEM micrographs confirm these findings, showing fewer cells attached to the pores of the control foam (Figure 9e). Furthermore, these cells are primarily balled up, with little to no distinction between nuclei and actin. The cell morphology on the bioactive foams indicates that these cells are happier and thriving—the cells are attached well and spreading on foam pores, forming cellular networks (Figure 9e), with an increase in cell spreading over the 72 h (Figure 9c). DAPI and phalloidin staining further confirm the increase in cell attachment and proliferation (nuclei, blue) and cell spreading (actin, red) over 72 h on the bioactive foams (Figure 10). Thus, the bioactive PUr SMP foams significantly improved cell interactions compared to the control PUr foam. Here, we studied NIH/3T3 mouse fibroblasts, a cell line commonly used in literature for initial cell analysis. Fibroblasts are a key cell in wound healing, responsible for creating and remodeling the ECM, signaling, and wound closure.^41^ Therefore, the increase in cell attachment, spreading, proliferation, and cell–cell interactions demonstrate the potential of these scaffolds to drive healing processes *in vivo*. Future studies will characterize cell interactions and healing processes with additional key wound healing cells and evaluate these processes in *in vivo* models of healing.

## 4 Discussion

Gelatin-based hemostatic dressings have many advantages: they trigger the activation and aggregation of platelets, enhance thrombin generation, and provide structural support for clot formation; gelatin-based dressings are nontoxic, nonantigenic, and have a high absorbing capacity; they are relatively low-cost and can be stored at room temperature.^42,43^ However, gelatin dressings are often limited by their poor mechanical strength, making them susceptible to tearing and disintegration under pressure and therefore incompatible with high-pressure bleeds.^44^ Additionally, gelatin sponges have significant swelling, which could result in tissue compression and potential damage.^45^ Gelfoam and Surgifoam are both absorbable gelatin sponges used for bleeding control that take about 4 to 6 weeks to dissolve completely.^46,47^ However, both commercially available treatment options liquefy within 2 to 5 days, making them unsuitable for structurally supporting new tissue.^47,48^

Collagen-based dressings similarly provide a physical matrix mimicking the natural ECM for the activation of platelets and trigger the coagulation cascade, leading to the release of thrombin and fibrinogen clotting factors.^49^ Collagen dressings offer good biocompatibility, cell adhesion, and can promote tissue regeneration.^50^ However, collagen has uncontrolled biodegradation and thermal instability.^51^ Helistat is a hemostatic collagen sponge used in dental and surgical procedures, but it is very expensive compared to traditional gauze.^52^ CollaStat is an injectable hemostatic agent requiring use by trained professionals, which is inconvenient for first-aid rescue.^53^ To that end, collagen and gelatin have been modified with synthetic polymers to improve their mechanical properties to produce hybrid cryogels and hydrogels.^54,55^ However, cryogels and hydrogels are limited in their ability to easily control pore size and morphology, maintain biocompatibility, and scale up production with consistent quality for clinical applications.^56,57^ Other recent advances in bioactive sponges and SMPs require lengthy modification, synthesis, and purification procedures that would similarly hinder clinical translation.^58,59^

PUr-based dressings have tunable degradation, unlike naturally derived biomaterials.^16,20,39^ PUr foams can maintain a moist wound environment because their high porosity and permeability allow for the transfer of moisture and oxygen; their mechanical strength allows them to withstand high-pressure environments, and they have high absorbency without causing compressive damage.^60,61^ PUrs are widely manufactured for use in construction, automative, and medical industries, emphasizing the feasibility of large-scale production.^62^ ResQFoam is a clinically available polyurethane hemostatic foam that uses a mechanical approach to stop bleeding by creating pressure against the injured tissue. It forms *in situ* by injection, but the foam-forming process is thermogenic, which can harm surrounding tissue.^63,64^ Injection and removal of ResQFoam also require experienced professionals, preventing use by a layperson.

Collagen- and gelatin-enhanced PUr SMP foam dressings have vast potential to improve upon the current clinically available treatment options. They have all the benefits of a synthetic SMP and the bioactivity of a collagen/gelatin dressing, without the many limitations of collagen- and gelatin-based dressings described above: The bioactive foams are absorbent, without excessive swelling; PUrs are inexpensive, easily scalable, and highly tunable; they are biocompatible, hemocompatible, and easily sterilizable; they can degrade within 3 months while still providing structural support for cell ingrowth and tissue regeneration; and their shape memory property offers ease of storage, delivery, and rapid expansion into deep, noncompressible, irregularly shaped wounds.^14,18,62^

Compared to similar PUr foams designed for wound hemostasis, the bioactive PUr foams explored in this work have increased platelet attachment and activation in both static and dynamic blood experiments (Figures 6b and 8b), which can ultimately improve clotting to stop bleeding faster.^39,65,66^ Other recent advancements in hybrid PUr scaffolds (e.g., PUr-gelatin scaffolds) rely on additional bioactive components like human amnion, soybean oil, and platelet-rich growth factor to improve cell-material interactions.^67−69^ The bioactive PUr foams developed here have comparable cell attachment and proliferation to these works without additional bioactive agents (Figures 9 and 10).

The primary advantage and novelty of this system is that the physical incorporation of gelatin and collagen into the PUr SMP foams is easily achieved within 1 h, allowing for a simple modification into a range of potential foam formulations without altering foam chemistry or foaming processes. The gelMA is also cross-linked postfoam fabrication, allowing for the chemical incorporation of gelatin without changing the overall foam chemistry. This process allows for ease of manufacturing and tunability of the dressings. When incorporated in small quantities (200 μg/mL), gelatin and collagen significantly improved blood and cell-material interactions (Figures 6–10), which could help reduce costs compared to gelatin- and collagen-based dressings, where the bioactive component is the primary material.

One potential limitation of the physical and chemical incorporation methods explored in this work is the long-term stability of the bioactive components, since they are not chemically cross-linked with the PUr foams. That said, the significant increase in cell attachment and spreading on the bioactive foams at 72 h suggests that even if the bioactive components dissolve partially, they are still highly effective at driving initial cell interactions on the foams. As the bioactive components dissolve and the foams degrade, the cells would leave behind their own ECM and replace the scaffolding. Future *in vivo* studies would provide valuable information on the stability and efficacy of the bioactive foams. Based on these *in vitro* findings, these foams are expected to stop hemorrhaging faster and improve wound epithelization, collagen deposition, and overall wound closure rates compared to the control foam.

## 5 Conclusions

The ease of fabricating the bioactive PUr SMP foams in this study can provide an affordable, effective hemostatic dressing that improves upon current treatment options. Incorporating the bioactive components into PUr foams maintained or improved their mechanical, thermal, and shape memory behavior compared to the control PUr. Collagen and gelatin increased platelet interactions, fibrin deposition, and clotting rates of the foams to improve their overall hemostatic effect. The bioactive components also significantly increased cell attachment, spreading, and proliferation on foam pores, which can help with the proliferation and remodeling stages of wound healing. Furthermore, the simple incorporation methods of collagen and gelatin offer broad tunability for various tissue engineering applications, where the PUr scaffold could be easily tailored to have different pore sizes, degradation rates, or thermal and mechanical properties based on the target tissue. Overall, this work offers a simple solution for improving the bioactivity of cell- and blood-contacting materials for wound healing applications.

## Supporting Information Available

Additional experimental details including stability of bioactive components on wet samples (FTIR), raw DSC data, and blank perfusion runs without samples (PDF)
