# Supplementary material for: Bioactive Polyurethane Shape Memory Polymer Foam Dressings with Enhanced Blood and Cell Interactions for Improved Wound Healing
Source: ACS Appl Mater Interfaces. 2025 Apr 22;17(18):26402–15. doi: 10.1021/acsami.5c02532 (PMC12067373; doi:10.1021/acsami.5c02532)
Supplement: Supplementary file 1 — am5c02532_si_001.pdf [file am5c02532_si_001.pdf]

Supporting Information

Bioactive polyurethane shape memory polymer foam dressings with enhanced blood and cell interactions for improved wound healing

Natalie Marie Petryk,[1] Nghia Le Ba Thai,[1] Leo Vikram Saldanha,[1] Shawn Tyrin Sutherland,[1] and Mary Beth B. Monroe[1]*

[1]Biomedical and Chemical Engineering and BioInspired Syracuse: Institute for Material and Living Systems, Syracuse University, Syracuse, New York, 13244

---

*Corresponding author:
Dr. Mary Beth Browning Monroe
Department of Biomedical and Chemical Engineering
Bioinspired Institute for Material and Living Systems
Syracuse University
318 Bowne Hall
Syracuse, NY 13244
Tel: (315) 443-3323
E-mail: mbmonroe@syr.edu

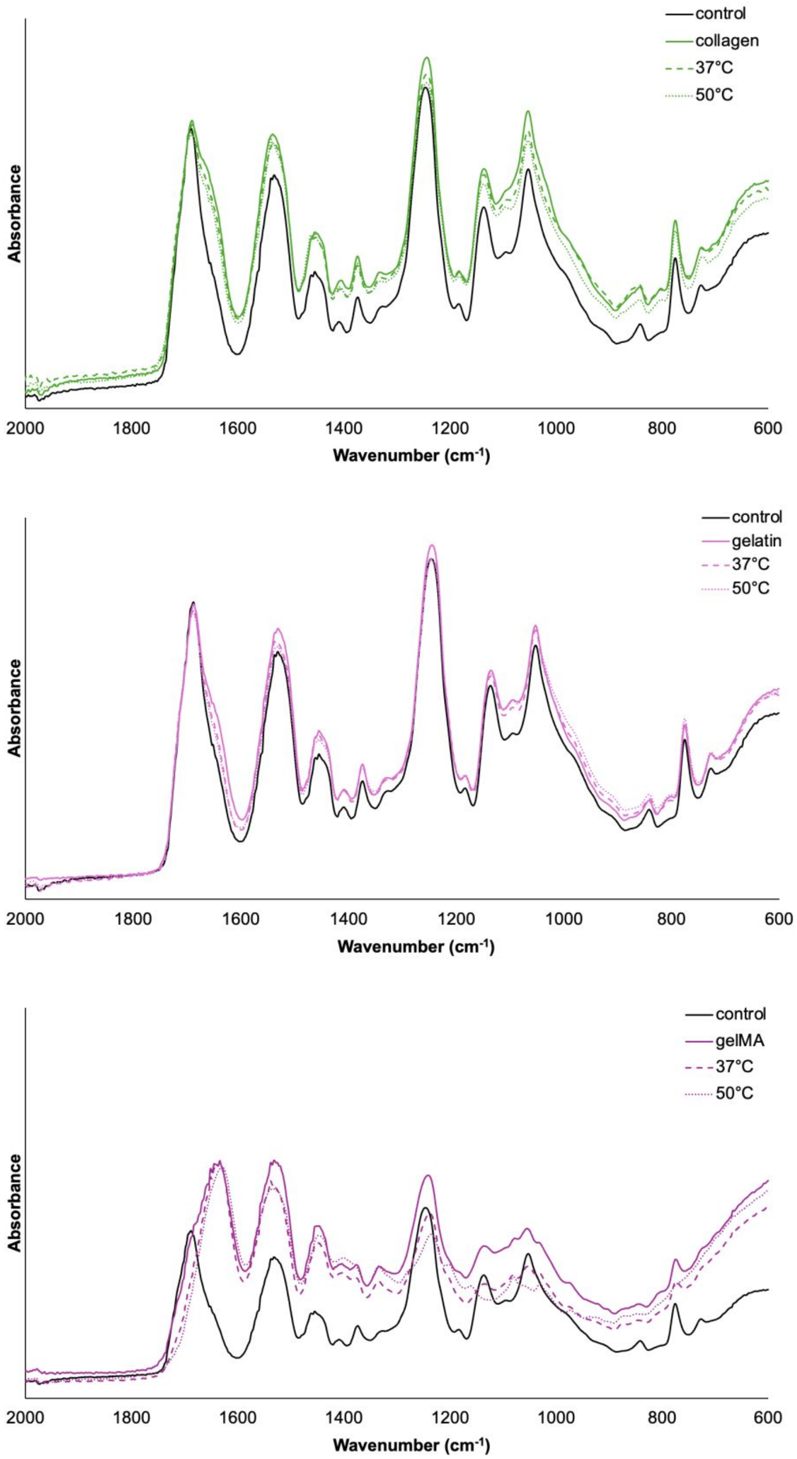

Figure S1. FTIR spectra comparing the bioactive foams before and after 1-hour incubation in DI water at 37°C and 50°C.

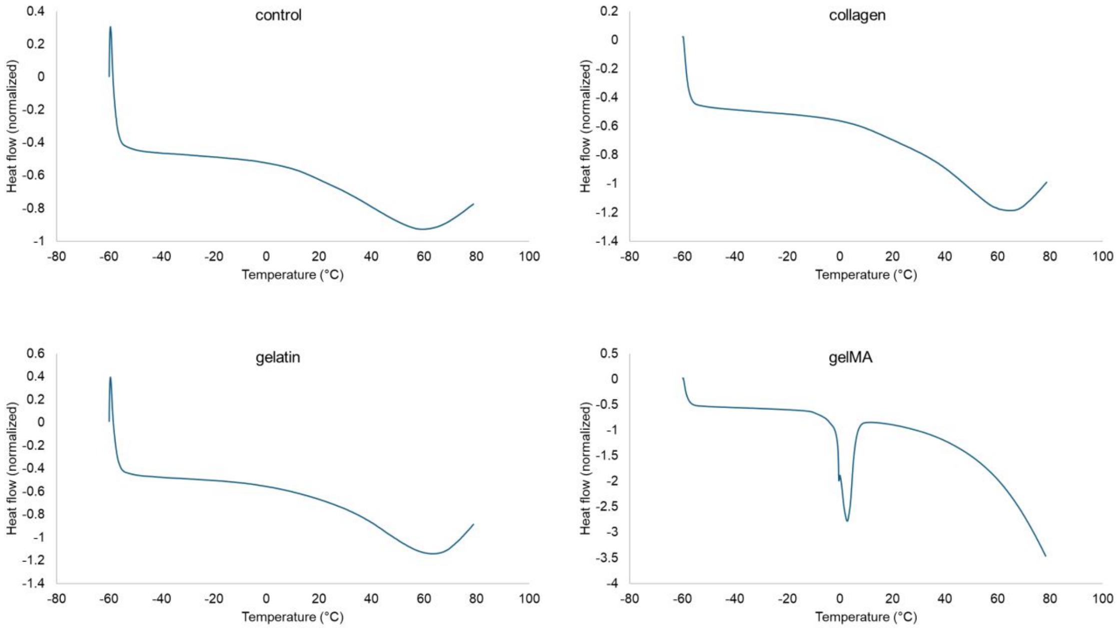

Figure S2. Representative DSC plots of wet (plasticized) samples.

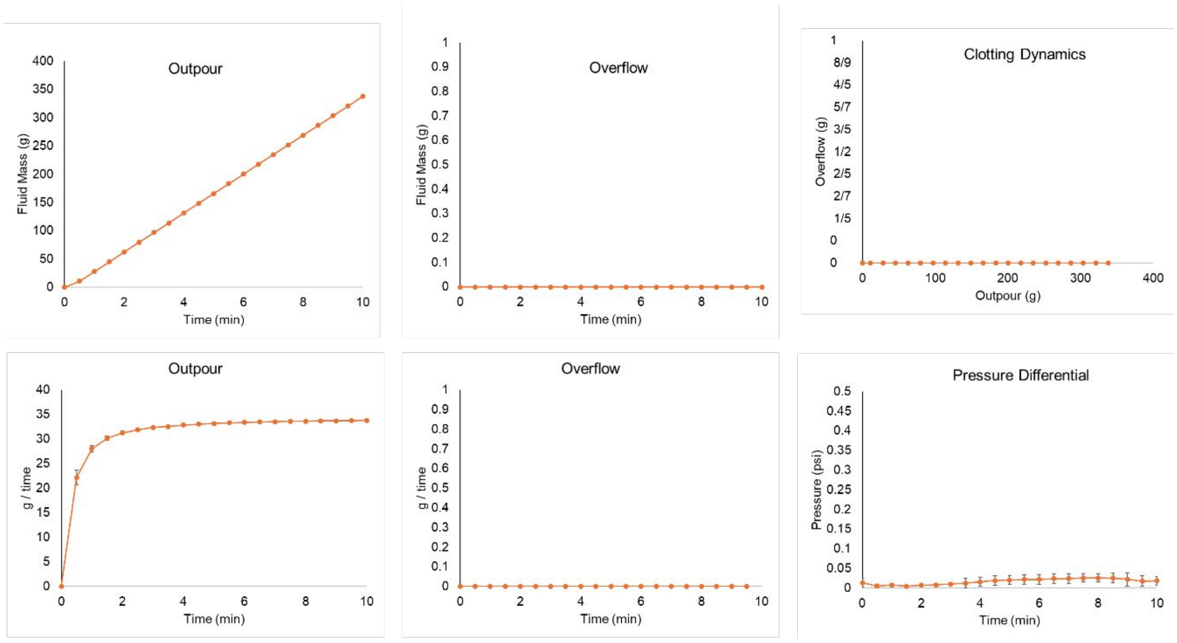

Figure S3. Standard perfusion runs (no sample) with Na-citrated whole porcine blood for 10 minutes.
